# Walnut (*Juglans regia* L.) Septum: Assessment of Bioactive Molecules and In Vitro Biological Effects

**DOI:** 10.3390/molecules25092187

**Published:** 2020-05-07

**Authors:** Marius Emil Rusu, Ionel Fizesan, Anca Pop, Andrei Mocan, Ana-Maria Gheldiu, Mihai Babota, Dan Cristian Vodnar, Ancuta Jurj, Ioana Berindan-Neagoe, Laurian Vlase, Daniela-Saveta Popa

**Affiliations:** 1 Department of Pharmaceutical Technology and Biopharmaceutics, Faculty of Pharmacy, Iuliu Hatieganu University of Medicine and Pharmacy, 8 Victor Babes Street, 400012 Cluj-Napoca, Romania; marius.e.rusu@gmail.com (M.E.R.); laurian.vlase@umfcluj.ro (L.V.); 2 Department of Toxicology, Faculty of Pharmacy, Iuliu Hatieganu University of Medicine and Pharmacy, 8 Victor Babes Street, 400012 Cluj-Napoca, Romania; anca.pop@umfcluj.ro (A.P.); dpopa@umfcluj.ro (D.-S.P.); 3 Department of Pharmaceutical Botany, Faculty of Pharmacy, Iuliu Hatieganu University of Medicine and Pharmacy, 8 Victor Babes Street, 400012 Cluj-Napoca, Romania; gheldiu.ana@umfcluj.ro (A.-M.G.); mihai.babota@umfcluj.ro (M.B.); 4 Department of Food Science, University of Agricultural Sciences and Veterinary Medicine, 3-5 Calea Manastur, 400372 Cluj-Napoca, Romania; dan.vodnar@usamvcluj.ro; 5 Research Center for Functional Genomics, Biomedicine and Translational Medicine, Iuliu Hatieganu University of Medicine and Pharmacy, 23 Marinescu Street, 400337 Cluj-Napoca, Romania; jurj.maria@umfcluj.ro (A.J.); ioana.neagoe@umfcluj.ro (I.B.-N.); 6 MEDFUTURE—Research Center for Advanced Medicine, Iuliu Hatieganu University of Medicine and Pharmacy, 23 Marinescu Street, 400337 Cluj-Napoca, Romania; 7 Department of Functional Genomics and Experimental Pathology, The Oncology Institute “Prof. Dr. Ion Chiricuta”, 34-36 Republicii Street, 400015 Cluj-Napoca, Romania

**Keywords:** walnut septum, tocopherols, LC-MS/MS, by-product, enzyme inhibitor, antimicrobial, antimutagenic, antioxidant, anti-inflammatory activity

## Abstract

Walnut (*Juglans regia* L.) septum represents an interesting bioactive compound source by-product. In our study, a rich phenolic walnut septum extract, previously selected, was further examined. The tocopherol content determined by liquid chromatography-tandem mass spectrometry (LC-MS/MS) revealed higher amounts of α-tocopherol compared to γ- and δ-tocopherols. Moreover, several biological activities were investigated. The in vitro inhibiting assessment against acetylcholinesterase, α-glucosidase, or lipase attested a real management potential in diabetes or obesity. The extract demonstrated very strong antimicrobial potential against *Staphylococcus aureus, Pseudomonas aeruginosa* and *Salmonella enteritidis*. It also revealed moderate (36.08%) and strong (43.27%) antimutagenic inhibitory effects against TA 98 and TA 100 strains. The cytotoxicity of the extract was assessed on cancerous (A549, T47D-KBluc, MCF-7) and normal (human gingival fibroblasts (HGF)) cell lines. Flow cytometry measurements confirmed the cytotoxicity of the extract in the cancerous cell lines. Additionally, the extract demonstrated antioxidant activity on all four cell types, as well as anti-inflammatory activity by lowering the inflammatory cytokines (interleukin-6 (IL-6), interleukin-8 (IL-8), interleukin-1 β (IL-1β)) evaluated in HGF cells. To the best of our knowledge, most of the cellular model analyses were performed for the first time in this matrix. The results prove that walnut septum may be a potential phytochemical source for pharmaceutical and food industry.

## 1. Introduction

Natural by-products generated in the food and agricultural processing industries, inexpensive and available in large quantities, are recognized as sources of active compounds having many different biological activities, such as antioxidant, anti-inflammatory, antimicrobial, or antimutagenic [1]. Increasing tendencies towards the use of natural ingredients instead of chemical preservatives have been noticed in the last decades.

Walnut (*Juglans regia* L.) is an important crop intended to produce nutritious nuts, key components of healthy diets correlated with quality lifespan [2]. In addition, some walnut by-products, such as leaves or green husks, were characterized and demonstrated to constitute good sources of bioactive molecules including tocopherols and phenolic compounds [3]. Walnut septum (WS), the membrane that divides the two halves of the walnut kernel, is another major by-product that revealed in vitro biological potential through its bioactive phytochemicals. In previous work we described the polyphenolic profile of a WS extract (WSE) obtained in the optimum extraction conditions for phenolic compounds and we demonstrated its high antioxidant capacity [4].

This study aimed to expand the knowledge regarding the WS phytochemical profile and to discover some new pharmacological effects in order to make the best use of it as a source of valuable bioactive compounds for food and pharmaceutical industries. Therefore, we planned to describe the phytochemical composition of *Juglans regia* L. septum by the tocopherol analysis and expand the discussion about the bioactive molecules in WS. Tocopherols are antioxidant lipophilic molecules that act as free radical scavengers, activate the Nuclear factor erythroid 2-related factor 2/Antioxidant Response Element (Nrf2/ARE) pathway [5], prevent lipid and cholesterol peroxidation, and suppress tumor angiogenesis [6]. They are important components of tree nuts, but there is no information so far related to their content in WS. Thus, the tocopherol content was determined in WSE by a new developed and validated liquid chromatographic method coupled with mass spectrometry in tandem (LC-MS/MS).

In the pharmacological screening step, natural products are currently examined as inhibitors of enzymes involved in physiological processes or various pathologies. The action of nuts or their by-product extracts against cholinesterase (neurodegenerative disorders), glucosidase (diabetes), or lipase (obesity) revealed encouraging results [7]. Since the effect of WS was previously investigated only on tyrosinase (an enzyme involved in skin wrinkles and aging) [4], we extended the analyses to determine the in vitro WSE effects on all the enzymes mentioned. Additionally, in vitro biological activities of WSE on different microorganisms and different cell lines were also examined. Thereby, the antibacterial (Gram-positive and Gram-negative bacteria), antifungal, and antimutagenic tests were performed. Moreover, the cytotoxic, antioxidant and anti-inflammatory effects were determined on three cancerous cell lines (the human lung adenocarcinoma A549, the human breast cancer T47D-KBluc and MCF-7) and one normal cell line (human gingival fibroblasts HGF) to assess an anticancerous potential.

Accordingly, this experiment brings important new information to complete the knowledge about WS and its therapeutic potential.

## 2. Results and Discussion

### 2.1. Bioactive Compounds Present in Walnut Septum

#### 2.1.1. Tocopherols

The tocopherol assay performed in this study completes the WS phytochemical profile previously described by our team in which the polyphenol content was determined [4]. In that study, we selected the optimum experimental conditions for obtaining an extract with the highest total phenolic content (TPC) and ABTS (2,2′-azino-bis(3-ethylbenzothiazoline)-6-sulphonic acid) antioxidant activity. Based on those conditions, in this study, we aimed to characterize the WSE tocopherol content and evaluate the degree to which tocopherols contribute to the biological antioxidant effects of the extract.

Vitamin E (VE), an essential fat-soluble vitamin, encompasses eight structurally similar compounds that include tocopherols and tocotrienols. As a powerful antioxidant and anti-inflammatory agent, VE can prevent low-density lipoprotein (LDL) cholesterol and lipid peroxidation via scavenging peroxyl radicals, enhance immune response, inhibit cell proliferation, or suppress tumor angiogenesis [6]. Based on the fact that VE shares common pathways as lipoproteins and cholesterol, pathophysiological conditions linked with lipid metabolism, such as obesity, could be negatively associated with VE status [8]. The European Food Safety Authority (EFSA) recognized that VE contributes to the protection of DNA, proteins and lipids from oxidative damage, and in the normal function of the immune system [9].

**Figure 1:**
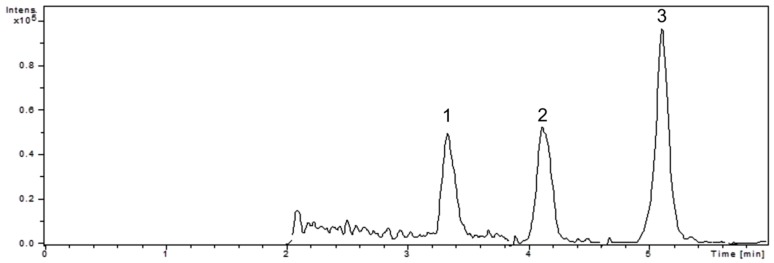
The chromatographic separation of tocopherols from walnut septum extract: (1) delta-tocopherol; (2) gamma-tocopherol; (3) alpha-tocopherol.

To separate and determine the tocopherol content from WSE, we developed and optimized a reliable and fast LC-MS/MS method with a run-time of 6 min and good resolution (Table 1). The chromatogram of *Juglans* septum extract is presented in Figure 1.

Since γ- and β-tocopherols have the same molar mass (416.7 g/mol) and, as mentioned by Kornsteiner et al. [10], cannot be separated by chromatography, we analyzed their total content and expressed them as γ/β-tocopherols. For a better and comparative evaluation of the results, the tocopherol content in walnut kernel extract (WE), from which the septum originated, was also determined (Table 2).

In comparison with the WE, the total tocopherol content in WSE was almost three times lower, 6.55 mg vs. 19.56 mg, due to the lower lipid content in this matrix. However, the quantity of α-tocopherol was more than four-fold in septum compared to walnut, representing 51.15% of the total tocopherol content in septum and only 3.99% of the total tocopherols in walnut. The WE tocopherol content in our study is comparable with those reported in previous walnut studies, with γ-tocopherol contributing the most to the total tocopherol content (17.3 to 26.2 mg/100 g), followed by α-tocopherol (0.87 to 1.66 mg/100 g) and δ-tocopherol (0.82 to 1.69 mg/100 g) [11]. Another experiment reported amounts of α-tocopherol between 0–0.8 mg/100 g, γ-tocopherol content varying from 12.6 to 26.7 mg/100 g, while δ-tocopherol ranged between 0.16–3.2 mg/100 g in walnuts, depending on the cultivar studied [12]. Other studies evaluated the presence of α-tocopherol and γ/β-tocopherols in almond and hazelnut oil [10]. In the two matrices, the α-tocopherol contents were 24.2 and 31.4 mg/100 g, respectively, while the γ/β-tocopherol contents were 3.1 and 6.9 mg/100 g, respectively, with no δ-tocopherol detected. The influence of walnut kernel maturity degree over the tocopherol and polyphenol contents was presented in the study of Pycia et al. [13]. The total tocopherol content increased with maturation and ranged from 8.25 to 18.30 mg/100g, depending on the cultivar, whereas the amount of phenolics decreased with the ripening of walnuts [13]. To the best of our knowledge, our study is the first quantification of the tocopherols in the septum of *Juglans regia* sp.

#### 2.1.2. Overview on Phenolic Compounds Identified in WSE and Their Biological Properties

In a previous study, we analyzed the phenolic compounds from WS extracts using an optimized liquid chromatography coupled with tandem mass spectrometry (LC-MS) [4]. The compounds were identified based on their MS spectra (molecular ion, *m/z*) and were further quantified by UV signal on the calibration curves built using standard compounds. From the 24 phenolic compounds analyzed, the following compounds were identified (limit of detection (LOD) > 0.1 µg/mL): gallic acid, protocatechuic acid, gentisic acid, chlorogenic acid, catechin, vanillic acid, syringic acid, epicatechin, p-coumaric, ferulic acid, hyperoside, isoquercitrin, and quercitrin, and nine of them could be quantified (limit of quantification (LOQ) > 0.5 µg/mL) (Table 3).

From the identified polyphenols, the ones with the highest concentration in WSE were the three quercetin glycosides and catechin. The measured quantities of quercitrin (quercetin-3-O-rhamnoside), isoquercitrin (quercetin 3-β-D-glucoside) and hyperoside (quercetin 3-D-galactoside) were 107.3, 10.36, and 6.73 mg, respectively (Table 3). Similar to our study, Liu et al. identified all three quercetin glycosides in the septum of *Juglans regia* cultivar ‘185′, one of the most planted cultivars due to its high-quality fruits [14]. However, a quantitative comparison between the results of that study and the results obtained in our experiment was not possible, due to the lack of quantitative data in the former study. Several other quercetin glycosides were identified in the leaves and green husks of walnut [15,16]. In comparison to our study where quercitrin had the highest concentration from the quercetin glycosides, in leaves the principal component was hyperoside, reaching 26.8% from the total phenolic content [15]. In addition to the quercetin glycosides, catechin, a polyphenol compound belonging to the flavonoid group, was also present in high quantities in the WSE. The presence of this flavonoid in the septum, leaves, and green husks was previously reported, however, due to the lack of quantitative data regarding the content in septum, no direct comparison was possible [14,16,17]. In contrast with walnut kernels, the catechin concentration was approximately 10 folds higher in the septum, reaching almost 60 mg/100g [17]. Other polyphenols, with a small molecular weight such as the gallic acid, syringic acid, and vanillic acid were also quantified in the WSE. The presence of these polyphenols was noticed in the hydroalcoholic extract of green immature walnuts, a drink used in folk medicine [16]. Similarly, the phenolic compounds described in septum were also found in walnut leaves, isoquercitrin being the main compound in that matrix [18].

Total phenolic content (TPC), total flavonoid and condensed tannin (proanthocyanidin) contents previously determined in the WSE (obtained in the same extraction conditions) were of 6,703 ± 976 mg gallic acid equivalents, 976 ± 23 mg quercetin equivalents, and 23,720 ± 322 mg catechin equivalents per 100 g septum, respectively, corresponding to an antioxidant activity (ABTS assay) of 16,862 ± 968 mg trolox equivalents per 100 g septum [4]. The TPC/tocopherol ratio in WSE is 343:1 and reflects a minor contribution of tocopherols to the antioxidant activity of WSE in relation to polyphenols. The total tocopherol content in walnut septum using specific extraction conditions for lipophilic molecules would be reevaluated in further analysis. Moreover, the analysis of hydrolyzable tannins, in particular ellagic acid and ellagitannins, specific components present in different parts of walnut (*Juglans regia* L.), with remarkable antioxidant properties, is a future research direction.

Nevertheless, the phenolic compounds, together with the tocopherols identified in the walnut septum and through their complex mechanisms of action, could be responsible and explain the biological potential of this walnut by-product.

### 2.2. Biological Activities

#### 2.2.1. Enzyme Inhibitory Activity

The in vitro action of nuts or their by-product extracts against acetylcholinesterase (treatment of cognitive signs related to neurodegenerative disorders) [19], amylase (treatment of type 2 diabetes mellitus (T2DM)) and lipase (management of obesity) [20], or tyrosinase [21] revealed encouraging results. Therefore, we investigated the enzymatic inhibitory activities of WSE on some key enzymes.

##### Cholinesterase Inhibitory Activity

As the cholinergic system was suggested to be the earliest and most affected system in neurodegenerative disorders, such as Alzheimer’s disease (AD), the scientific community started examining the use of cholinesterase inhibitors as therapeutic agents that can increase cholinergic activity, enhance cognition and cognitive performance, and lessen psychiatric disturbances. Among cholinesterases, acetylcholinesterase (AChE) controls cholinergic nerve and chemical transmission by hydrolyzing the neurotransmitter acetylcholine.

In our experiment, only the 2 mg/mL concentration extract revealed a very weak (0.68%) inhibitory activity. Thus, we can conclude that WS has no AChE inhibitory potential. Our literature search found only one study that presented AChE inhibitory activity of walnut flowers and leaves [22]. According to that study, various types of polyphenols in the extracts could be responsible for the inhibiting effects. However, another study, in line with our results, found nine extracts of walnut fruits and leaves to be ineffective against acetylcholinesterase activity [23], stressing that the inhibitory activity may be dependent on the extraction methods used, or solvents and concentrations applied.

##### α-Glucosidase Inhibitory Activity

The control of postprandial hyperglycemia, an important step in preventing obesity and T2DM, and the development of atherosclerosis and cardiovascular diseases, is typically accomplished using various amylase or α-glucosidase inhibitors. α-Amylase is part of the first step in carbohydrate metabolism, transforming starch to oligosaccharides, while α-glucosidase further hydrolyzes oligosaccharides to glucose. The resulting glucose is absorbed into the small intestine and the blood glycemia is increased [24].

Studies demonstrated that proteins and polyphenols extracted from nuts, vegetables, and cereals can inhibit dietary carbohydrate absorption and aid in the treatment of diabetes [25]. The same trend was seen in hazelnut involucre, another nut by-product [26]. In this study, the IC_50_ for the 50% aqueous acetone septum extract was 0.14 ± 0.02 mg/mL, a much stronger potential than acarbose (IC_50_ = 0.80 mg/mL), the drug used in the treatment of T2DM. Thus, these results proved that bioactive compounds found in WSE can obstruct the activity of intestinal α-glucosidase, thus slowing the starch metabolism. Moreover, this evidence is in full agreement with other studies on the antidiabetic potential of walnut septum extracts [27] and it offers scientific basis on the nutraceutical potential of the WSE in the management of diabetes.

##### Lipase Inhibitory Activity

Obesity, a worldwide major health issue, generated by an imbalance between energy intake and expenditure, was linked to many cardiometabolic diseases, leading morbidity and mortality causes. Among the therapies against obesity, the inhibition of pancreatic lipase, an important enzyme involved in the hydrolysis of dietary fats, digestion of triglycerides, and lipid absorption is one of the main researched mechanisms. Therefore, lipase inhibition in the digestive system is an effective method to prevent the progress of obesity. Since orlistat, the clinically approved drug used in the treatment of obesity could display serious side effects, including headache, increased blood pressure, constipation, or insomnia [28], WS has been assayed for its obesity treatment potential.

Our results reveal that the WSE, at the concentration of 1 mg/mL, has a relative inhibition capacity of 50.79% from that of orlistat, the positive control. Based on previous findings that phenol compounds from walnut kernels inhibited lipase activity in vitro [29], we argue that the phenolic acids and flavonoids identified in WSE could be part of the lipase inhibition mechanism.

#### 2.2.2. Antibacterial and Antifungal Activity

The assessments of antibacterial and antifungal potential of WSE are presented in Table 4.

The effects were measured by microdilution assays against a panel of different Gram-positive (*Staphylococcus aureus*) and Gram-negative (*Escherichia coli*, *Pseudomonas aeruginosa*, *Salmonella enteritidis*) bacteria, and two fungi (*Candida albicans* and *Candida parapsilosis*), selected based on their relevance for public health. To the best of our knowledge, there are no scientific reports concerning the bacterial or antifungal inhibitory properties for this plant matrix. The WSE had minimum inhibitory concentration (MIC) levels between 0.012–3.12 mg/mL. The best antibacterial effect was obtained against *P. aeruginosa* (MIC = 0.012 mg/mL), while the same values were detected against *S. aureus* and *S. enteritidis* (MIC = 0.098 mg/mL). The lowest effect was noticed against *E. coli*.

As previously reported [30], MIC values of less than 0.5 mg/mL suggest good antibacterial activity. Based on this report, WSE demonstrated very strong antimicrobial potential against three of the bacterial strains. The antimicrobial activity of *Juglans* septum can be attributed to its content of polyphenols, especially flavonoids (e.g., catechin, epicatechin), quercetin glycosides (hyperoside, isoquercitrin, quercitrin), and condensed tannins [4]. As known, the active molecules have to pass the lipopolysaccharide outer membrane and then the inner cell membrane, in the case of Gram-negative bacteria. Thus, the antimicrobial effect is correlated to the ability of the compounds to penetrate the membranes and reach the site of action. Concerning the antifungal activity, both fungal strains presented low sensitivity for the septum extract. Our results are similar with previous findings obtained for other walnut by-products. In those studies, fungal species also showed resistance for the action of walnut leaves and green husks, while only green husk extracts revealed activity against *P. aeruginosa* [15,31].

#### 2.2.3. Antimutagenic Assay

Previous investigations suggested that antimutagens from natural dietary sources, such as phenolic compounds, might play essential roles in the antimutagenic activity of vegetables or fruits and could be effective in preventing genetic diseases or cancer [32]. The Ames test is a bacterial reverse mutation assay designed to identify new molecules with mutagenic potential. The assay uses histidine-dependent *Salmonella* strains with pre-existing mutations that leave the bacteria unable to form colonies in the absence of histidine. New mutations at the site of these pre-existing mutations may restore the function of the genes and let the cells to synthesize histidine. Only bacteria that revert to histidine independence can form colonies. Based on a previous study [33], the antimutagenic effect was considered strong when the inhibitory effect of extracts was >40%, moderate when the inhibitory effect was in the range of 25–40%, and an inhibitory effect <25% was recognized as weak, and it was not considered a positive result. In our study, WSE showed moderate (36.08%) and strong (43.27%) antimutagenic inhibitory effects against TA 98 and TA 100 strains, respectively (Table 5). As flavonoids have been suggested as antigenotoxic agents [34], we argue that the antimutagenic activity of the extracts is linked to the phenolic compound content and flavonoid glycosides.

#### 2.2.4. Cytocompatibility

**Figure 2:**
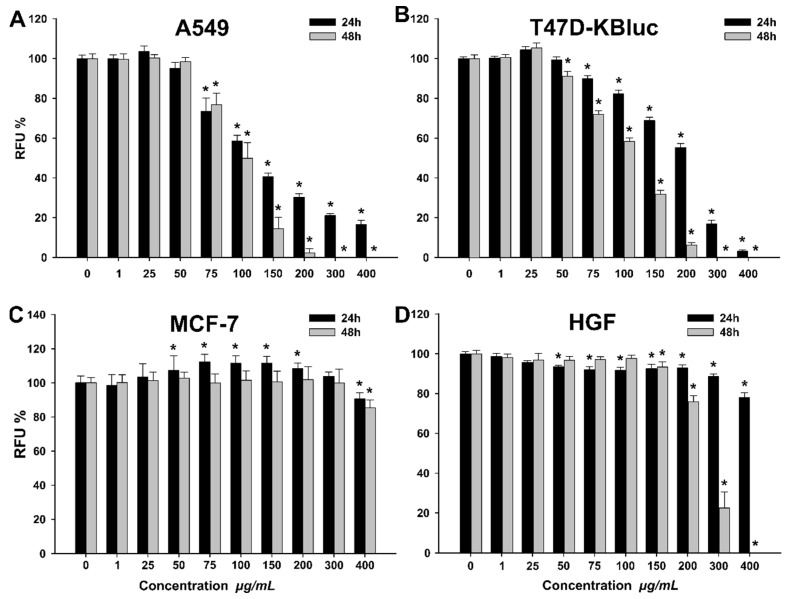
Cytotoxic effect of the septum extract observed using Alamar Blue assay on A549 (**A**), T47D-KBluc (**B**), MCF-7(**C**) and human gingival fibroblasts (HGF) (**D**). The results are expressed as relative means ± standard deviations (six technical replicates for each of the three biological replicates) where the negative control (DMSO 0.2%) is 100%. Asterisks (*) indicate significant differences (*p* < 0.05) compared to the negative control. RFU— relative fluorescence units.

Exposure of cancerous A549 and T47D-KBluc cells to WSE resulted in a dose-dependent decrease in the cellular viability (Figure 2). In comparison with cells exposed only to cellular media, the vehicle (cellular media + 0.2% DMSO) did not affect the cellular viability (data not shown).

A higher cytotoxic effect was observed after 48 h incubation, in comparison with the 24 h incubation. The calculated IC_50_ values for A549 were 80.02 ± 4.33 and 70.79 ± 1.93 µg/mL WSE at 24 h and 48 h, respectively (Table 6).

A similar profile with more pronounced cytotoxicity at 48 h was observed for T47D-KBluc. The calculated IC_50_ values were 265.60 ± 53.79 and 112.75 ± 6.38 µg/mL, at 24 h and 48 h, respectively (Table 6). In order to elucidate the cellular death induced by WSE exposure in A549 and T47D-KBluc cell types, the ratio between viable, apoptotic and necrotic cells was evaluated using flow cytometry. Flow cytometry measurements confirmed the results obtained using Alamar Blue assay, the WSE exposure inducing a dose-dependent cytotoxicity in the cell lines evaluated (Table 7).

In addition, the flow cytometry results pinpoint that the mechanism of cellular death is mainly necrosis, apoptosis having a minor contribution only at intermediary doses (Table 7). In comparison with the A549 and T47D-KBluc cell lines, the normal HGF cell type was more resilient to the cytotoxic potential of the WSE. Exposure of HGF cells to the highest tested dose of 400 µg/mL resulted in mild cytotoxicity with a decrease in viability to approximately 80% at 24 h (Figure 2). These results are in agreement with data published by other research groups that evaluated the potential anticancerous effects of different extracts from the seeds, leaves, bark and the green husks of *Juglans regia* [3,35,36]. Santos et al. reported that the methanolic extract of walnut leaves induced cellular death in cancerous cells in a dose-dependent manner, while at similar doses no toxicity was observed on non-tumor primary hepatic cells [3]. Moreover, the reported IC_50_ values were similar to the ones presented in the current study, varying from 200 to 400 µg/mL depending on the cancerous cell line used [3]. Interestingly, in our study the cancerous cell line MCF-7 displayed a particular behavior when exposed to the WSE. A cytotoxic effect was observed only at the highest tested dose of 400 µg/mL, where the cellular viability decreased at approximately 80% at both time points. Moreover, a hormetic effect was observed at the intermediary concentrations where a statistically significant increase in viability over the negative control was noticed. We hypothesize that due to the higher resilience of the MCF-7 cell type, the non-toxic doses of the WSE increased the cellular metabolism, the biological function which was evaluated in the current study by Alamar Blue assay.

The anticancerous properties of the *Juglans regia* extracts have been associated with the presence of bioactive compounds such as phenolic compounds or tocopherols [37]. The extract evaluated in the current study was selected based on the high phenolic content [4], but as reported in the present study, it also contains tocopherols, which were not previously described in the case of WS. A recent long-term prospective cohort study concluded that higher α-tocopherol serum level was significantly linked with lower all-cause mortality [6]. However, preclinical studies exposed that γ- and δ-tocopherol are stronger than α-tocopherol in blocking the proliferation of cancer cells via inhibiting multiple cancer-promoting pathways, such as cyclo-oxygenase (COX) and 5-lipoxygenase (5-LOX), and transcription factors including Nuclear factor-κB (NF-κB) [38]. Similarly, only γ-tocopherol level was linked to telomere length, hence biological aging increase [39]. Nevertheless, scientific findings demonstrated that all VE isoforms could limit the progression of diseases, which may be due to the synergistic actions between VE and other micronutrients [40].

One problem that could arise in the case of walnut septum, compared to walnut kernel, could be the bioaccessibility and bioavailability of tocopherols, therefore a future research direction would be bioaccessibility/bioavailability studies of the bioactive compounds in WS, both in vitro and in vivo.

#### 2.2.5. Antioxidant Potential

**Figure 3:**
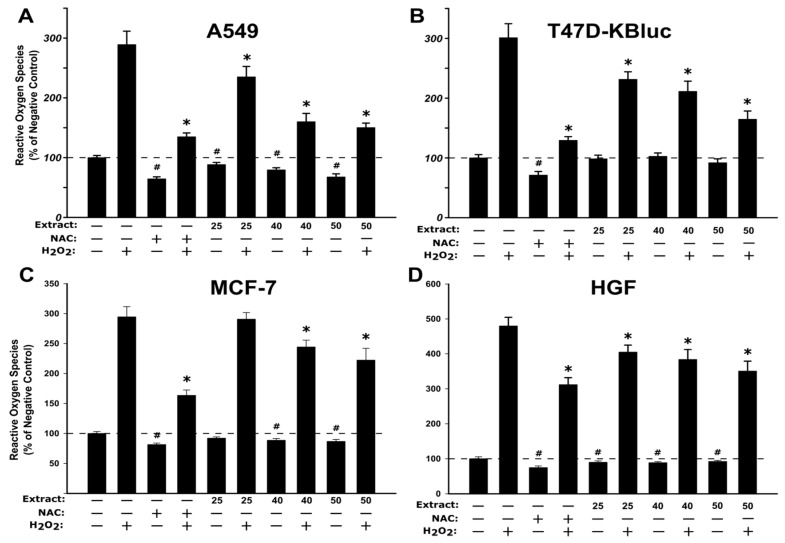
Antioxidant effect of the WSE evaluated using DCFH-DA (2ʹ,7ʹ-Dichlorofluorescin Diacetate) assay on A549 (**A**), T47D-KBluc (**B**), MCF-7(**C**) and HGF (**D**). The cellular model was pre-exposed to the extract (25, 40, and 50 µg/mL) or N-acetylcysteine (NAC) (20 mM) for 24 h, and further incubated with 50 µM DCFH-DA. The antioxidant effect of the WSE was evaluated after 2 h in stimulated (250 µM H_2_O_2_) and un-stimulated conditions. The results are expressed as relative means ± standard deviations (six technical replicates for each of the three biological replicates) where the negative control (DMSO 0.2%) is 100%. The asterisks (*) indicate significant differences compared to the positive control (250 µM H_2_O_2_) in stimulated conditions, while (#) indicate significant differences compared to the negative control in non-stimulated conditions (ANOVA + Tukey; *p* < 0.05).

The antioxidant effect of the WSE was evaluated on all four cell types at three concentrations (25, 40, 50 µg/mL) that did not affect the cellular viability (Figure 2). In the non-stimulated condition, exposure to the extract alone decreased in a dose-dependent manner the quantity of reactive oxygen species (ROS) in A549, MCF-7 and HGF cell types (Figure 3). Similar to the observations made for the extract, treatment with 20 mM N-acetylcysteine (NAC) reduced significantly the ROS in non-stimulated condition. In stimulated conditions, H_2_O_2_ exposure resulted in an increase of ROS that was partially inhibited by pre-treatment with NAC in all cell types (Figure 3). Exposure to the WSE resulted in a dose-dependent decrease in ROS in stimulated conditions, the highest concentrations tested displaying an antioxidant potential almost equal to the one exerted by NAC (Figure 3).

The results obtained indicate an antioxidant potential in in vitro cell cultures and are in agreement with other studies where the antioxidant potential of different extracts from *Juglans regia* was evaluated [41,42]. Based on the same assay as used in the current study, Muzzafer et al. reported that the methanolic extract of male flower of *Juglans regia* reduced the oxidative stress induced by UV exposure in HaCaT cells [42], while Muthaiyah et al. reported a protective effect of walnut extract against amyloid beta peptide-induced oxidative stress in PC12 cells [41]. The current results are also supported by studies that reported the antioxidant potential of extracts from different parts of the *Juglans regia* sp. in non-cellular assays such as FRAP (ferric reducing antioxidant power), DPPH (2,2-diphenyl-1-(2,4,6-trinitro-phenyl) hydrazine) and TEAC (trolox equivalent antioxidant capacity) [36,43].

In addition to the phenolic content of WSE, tocopherols act as strong antioxidants, with δ- and γ-tocopherol more effectively trapping the reactive nitrogen species (RNS) than α-tocopherol [44]. Tocopherols, as well as their corresponding tocotrienols, are very important lipophilic radical-scavenging antioxidants that break the lipid peroxidation cycle. Exposure to α-tocopherol reduced the concentration of ROS in cultured human bronchial epithelial cells pre-exposed to acrolein, a highly reactive unsaturated hazardous air pollutant [45]. Moreover, exposure to α-tocopherol enhanced the recovery of cellular glutathione level, depleted after acrolein exposure [45], pinpointing an indirect antioxidant mechanism. In a similar study, supplementation with α-tocopherol reduced the oxidative stress and the cytotoxicity of emamectin benzoate, a common pesticide [46]. More than several studies revealed that tocopherols, as well as polyphenols, have an indirect antioxidant potential by activating the Nrf2/ARE pathway that can initiate the synthesis of cellular antioxidants such as glutathione [47,48].

Other compounds present in *Juglans regia*, such as quercetin and quercetin glycosides, have been shown to possess antioxidant potential in in vitro and in vivo studies [49]. The antioxidant potential of quercetin aglycon and quercetin 3-*O-*β-D glucuronide (Q3GA), the main metabolite present in human plasma was previously reported in mouse fibroblasts [50]. Co-exposure to H_2_O_2_ and quercetin or Q3GA reduced the quantity of ROS, measured using the DCFH-DA (2ʹ,7ʹ-Dichlorofluorescin Diacetate) assay, at concentrations of 1 and 10 µM [50]. In addition, the antioxidant potential of quercitrin and isoquercitrin was reported in human hepatocytes where the co-exposure to these glycosides with several pro-oxidants (2,2’-Azobis(2-amidinopropane) dihydrochloride (AAPH), Cu^2+^ and H_2_O_2_) significantly reduced ROS [51]. In addition to the direct antioxidant effect, quercetin has been shown to up-regulate the Nrf2/ARE pathway through the regulation of both transcription and post-transcription sites and by repression of the Keap1 protein that suppresses the nuclear translocation of Nrf2 [52]. In the downstream of the Nrf2/ARE pathway, quercetin has been shown to up-regulate the expression of heme-oxygenase 1 (HO-1), up-regulation that partially abrogated the ethanol-induced oxidative stress in human hepatocytes [53]. Based on similar antioxidant mechanisms, exposure to hyperoside, another quercetin glycoside quantified in WSE in the current study, has been shown to induce the Nrf2 nuclear translocation and the mRNA and protein expression of HO-1 in human lens epithelial cells [54].

#### 2.2.6. Anti-Inflammatory Potential

**Figure 4:**
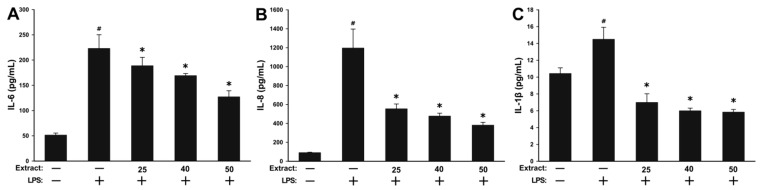
The extracellular release of pro-inflammatory cytokines interleukin-6 (IL-6) (**A**), interleukin-8 (IL-8) (**B**) and interleukin-1 β (IL-1β) (**C**) was analyzed in cell-free supernatants by ELISA at 24 h post-exposure to three concentrations of the walnut septum extract (WSE) in combination with 100 ng/mL LPS (lipopolysaccharides). The values are expressed as mean ± standard deviation (SD) of four biological replicates. The asterisk (*) indicates significant differences compared to the positive control (100 ng/mL LPS), while (#) indicates a significant difference of the positive control compared to the negative control (ANOVA + Tukey; *p* < 0.05).

In light of the promising results obtained in the antioxidant assay and the composition of WSE that included compounds with known anti-inflammatory potential such as tocopherols and polyphenols, the ability of WSE to inhibit induced inflammation in normal cells (HGF) was evaluated. Prior to the testing, the cellular viability was evaluated for a concomitant exposure of 24 h to 100 ng/mL LPS (lipopolysaccharides) and the three concentrations of the extract that were previously selected due to their absence of cytotoxicity, with no additive/synergic toxicity being observed (data not shown). Similar to the above-mentioned experiment, the ability of WSE to mitigate inflammation in LPS-stimulated HGF was evaluated by measuring the levels of interleukin-6 (IL-6), interleukin-8 (IL-8) and interleukin-1 β (IL-1β) from the cell-free culture supernatants. Exposure of cells to 100 ng/mL LPS induced a potent inflammatory response, the level of the inflammatory cytokines increasing by approximately 4.4-, 13.1- and 1.4-fold for IL-6, IL-8 and IL-1β, respectively (Figure 4). Statistical significance between the negative control and the positive control was observed for all the cytokines evaluated indicating that the inflammatory model was well designed. Co-exposure to the WSE resulted in a dose-dependent decrease in the levels of all three inflammatory cytokines evaluated. At the highest tested dose of 50 µg/mL, the WSE reduced the LPS-induced secretion of IL-6 by 55% and of IL-8 by 75%. Interestingly, the measured levels of IL-1β after co-exposure to the WSE at all doses were considerably lower than in the negative control condition, the highest dose of extract reducing the LPS-induced secretion of IL-1β by 170% (Figure 4).

In addition to inducing the antioxidant defense cellular systems, the activation of the Nrf2 transcription factor by phytochemicals present in walnuts and walnut by-products is inhibiting the activation of the NF-κB pathway and the resulting pro-inflammatory cascade [55]. The activation and nuclear translocation of NF-κB is associated with the synthesis of pro-inflammatory cytokines (IL-6, IL-8, IL-1β, etc), chemokines (Monocyte Chemoattractant Protein-1(MCP-1), Macrophage Inflammatory Protein-1 (MIP-1)) and enzymes (Cyclooxygenase (COX), Inducible Nitric Oxide Synthetase (iNOS)). The hydroethanolic extract of *Juglans regia* green husks has been shown to mitigate nitric oxide (NO) production in immune cells (RAW264.7) in response to LPS stimulation. Regarding the phytochemical profile of the above-mentioned extract, the authors reported the presence of phenolic acids, flavonoids, as well as tetralone and naphthalene derivatives, compounds usually described in the phytochemical profile of walnut [56]. Similarly, pretreatment of HaCaT cells with a methanolic extract of male flowers of walnut significantly prevented the secretion of ultraviolet B (UVB) activated inflammatory markers like IL-1, IL-6, tumor necrosis factor- α (TNF-α) and COX-2 [42]. The anti-inflammatory potential of extracts derived from *Juglans regia* was also reported in isolated human aortic endothelial cells, where co-exposure to the plant extract and the pro-inflammatory stimulus (TNF-α) reduced the expression of Vascular Cell Adhesion Molecule-1 (VCAM-1) and Intercellular Adhesion Molecule-1 (ICAM-1), two important pro-inflammatory adhesion molecules [57]. The observed data in vitro are supported by data reported in in vivo and human studies [58,59]. Qamar et al. reported that the walnut kernel extract modulates the acute inflammation, oxidative stress and lung injury in Wistar rats exposed to cigarette smoke extract [60], while Hosseinzadeh et al. reported an antinociceptive and anti-inflammatory effect of leaves extract in mice [61]. Contrary to the results reported for the total alcoholic extracts from various parts of *Juglans regia*, extracted and purified polysaccharides from the septum of walnuts have been shown to enhance macrophage functions and phagocytosis and stimulate the production of NO, TNF-α, IL-6, and IL-8 in RAW264.7 macrophage cells [62]. The increase in these pro-inflammatory and immune-stimulating mediators was mediated by several membrane receptors, including the Toll-like Receptor 2 (TLR2), and was accompanied by an increase in their mRNA levels, indicating an increased nuclear transcription of these mediators [62].

## 3. Materials and Methods

### 3.1. Reagents

All reagents and standards used were of analytical grade. Reference standards of α-tocopherol (≥95.5%) and γ-tocopherol (≥96% for high-performance liquid chromatography (HPLC)) were purchased from Sigma-Aldrich (Schnelldorf, Germany), and δ-tocopherol from Supelco (Bellefonte, PA, USA). Acetone and methanol of HPLC analytical-grade were acquired from Merck (Darmstadt, Germany) and dimethyl sulfoxide (DMSO ≥ 99.5%) from Riedel-de Haën (Seelze, Germany). The following reagents: 5,5-dithio-bis(2-nitrobenzoic) acid (DTNB), disodium hydrogen phosphate, potassium dihydrogen phosphate, acetylthiocholine iodide (ATCI), glutathione, p-nitrophenyl-α-D-glucopyranoside (PNPG), p-nitrophenyl-butyrate (PNPB), sodium carbonate, sodium chloride, 4-nitro-o-phenylenediamine (4-NPD), sodium azide (NaN_3_), 2ʹ,7ʹ-Dichlorofluorescin Diacetate (DCFH-DA), galantamine, acarbose, orlistat, gentamycin, fluconazole, N-acetyl cysteine (NAC), propidium iodide, trypsin, and hydrogen peroxide were bought from Sigma-Aldrich (Schnelldorf, Germany). Resazurin, insulin (cell culture tested), L-glutamine, sodium pyruvate, fetal bovine serum (FBS), phenol-red free Dulbecco’s modified Eagle’s medium F-12(DMEM/F-12) and MEM non-essential amino acid solution were all obtained from Sigma-Aldrich (Steinheim, Germany). Tris (tris(hydroxymethyl)-aminomethane) was acquired from Fluka (Buchs, Switzerland) and hydrochloric acid (37%) from Merck (Darmstadt, Germany). Dulbecco’s phosphate-buffered saline (PBS) was purchased from Invitrogen-Life Technologies (Darmstadt, Germany). The water used in our study was ultrapure obtained from a Milli-Q ultrapure water system (Millipore).

### 3.2. Plant Matrices

WS was obtained from walnuts harvested in Buciumi, Maramures County (47°28′ N, 23°29′ E), Northern part of Romania, in the fall of 2018. The optimal extraction conditions used to prepare the richest polyphenolic extract, the equipment and methods employed for the experiment were previously presented [4]. In brief, WS was mixed with water/acetone (50:50, *v/v*) at a ratio of 1:10 (*w/v*). The extraction was performed by Ultra-Turrax (T 18; IKA Labortechnik, Staufen, Germany) (2 min) followed by agitation using a vortex (RX-3, Velp Scientifica, Usmate, Italy) (2 min). After centrifugation (3000 rpm, 15 min), the supernatant was carefully separated, acetone removed using a rotary evaporator (Hei-VAP, Heidolph Instruments GmbH & Co., Schwabach, Germany), and the extract lyophilized (Advantage 2.0, SP Scientific, Warminster, PA, USA). In all the biological tests performed we used the lyophilized WSE kept in the dark at 4 °C until the determinations. If not mentioned otherwise, prior to analyses an appropriate amount of lyophilized WSE was solubilized in 5% DMSO and diluted with Milli-Q water.

An extract from walnut kernels with skin, in the same extraction conditions as above, was also prepared. This extract (10 μL supernatant obtained after centrifugation) was analyzed by direct injection into the LC-MS/MS system.

All assays were executed in triplicate.

### 3.3. Tocopherol Quantification by LC-MS/MS

#### 3.3.1. Chromatographic Analysis

The tocopherol content in the vegetal matrix was analyzed by a new developed and validated high-throughput LC-MS/MS method. The HPLC system was an Agilent 1100 series (binary pump, autosampler, thermostat; Agilent Technologies, Santa Clara, CA, USA), coupled with a Brucker Ion Trap SL (Brucker Daltonics GmbH, Leipzig, Germany). A Zorbax SB-C18 chromatographic column (100 × 3.0 mm i.d., 3.5 μm; Agilent Technologies, Santa Clara, CA, USA) was used. The mobile phase consisted of 7:93 (*v/v*) water/methanol, with isocratic elution. The flow rate was 1 mL/min and the thermostat temperature set at 40 °C. The mass spectrometry detection was performed in negative mode using an atmospheric pressure chemical ionization (APCI) source and multiple-reaction monitoring (MRM) mode. A volume of 10 μL of standard solution or extract was injected into the liquid chromatography/mass spectrometry system.

#### 3.3.2. Sample Preparation

Stock standard solutions (1 mg/mL) were prepared from α-tocopherol, γ-tocopherol, and δ-tocopherol in methanol and working solutions were obtained by appropriate dilutions in water/acetone (50:50, *v/v*). The calibration curves were linear in the concentration range of 40 to 960 ng/mL for all determined tocopherols, with a correlation coefficient r greater than 0.99.

For tocopherol quantification in plant matrices (both in WSE and walnut kernel (WK) with skin), the extracts (10 μL of each supernatant obtained after centrifugation) were analyzed by direct injection into the LC-MS/MS system.

### 3.4. Enzyme Inhibitory Activity

#### 3.4.1. Cholinesterase Inhibition Assay

The potential of WSE to inhibit AChE activity was measured using the Ellman’s method as described before [63], with slight modifications. A plant extract stock solution (2 mg/mL) in buffer A (50 mM Tris–HCl, pH 8) was used, from which different solutions were prepared with the following concentrations: 125, 250, 500, 750, and 1000 µg/mL. First, either 25 μL WSE in buffer A or buffer A, 25 μL of 15 mM acetylthiocholine iodide (ATCI) in Millipore water, 50 μL buffer B (50 mM Tris–HCl, pH 8, 0.1% fetal bovine serum), and either 25 μL AChE (Electric eel acetylcholinesterase, Type-VI- S, EC 3.1.1.7, Sigma-Aldrich) or buffer B were incubated in a 96-well plate for 15 min at 25 °C. Then, 125 μL of 3 mM DTNB in buffer C (50 mM Tris–HCl, pH 8, 0.1 M NaCl, 0.02 M MgCl_2_ 6H_2_O) were added and incubated again for 15 min at 25 °C. The absorbance was measured at 405 nm using an ELISA reader (SPECTROstar Nano Multi—Detection Microplate Reader, BMG Labtech, Ortenberg, Germany). Galantamine, a known AChE inhibitor, was used as reference substance for the positive control.

The AChE was expressed as inhibition percentage according to the Equation (1):

(1) Inhibition (%) = 100 − (*B − b*)/(*A − a*) × 100

where *A* is the activity without inhibitor, *a* is the negative control without inhibitor, *B* is the activity with inhibitor, and *b* is the negative control with inhibitor.

#### 3.4.2. α-Glucosidase Inhibition Assay

The inhibitory activity of α-glucosidase was tested using a protocol previously described [24]. Briefly, the sample solution (50 µL), glutathione (50 µL), α-glucosidase solution (50 µL) in phosphate buffer (pH 6.8), and p-nitrophenyl-β-D-glucuronide (PNPG) solution (50 µL) were mixed in a 96-well microplate and incubated at 37 °C for 15 min. The blank was similarly prepared but without the enzyme solution. After the incubation, the reaction was stopped by adding sodium carbonate (50 µL, 0.2 M). The absorbance was measured at 400 nm and the α-glucosidase inhibitory activity (%) was compared to the inhibitory activity of acarbose as reference substance.

#### 3.4.3. Lipase Inhibition Assay

The lipase inhibitory activity was determined by measuring the hydrolysis of p-nitrophenyl butyrate (p-NPB) to p-nitrophenol using a slightly modified reported method [64]. Briefly, 40 µL WSE and 40 μL pancreatic lipase (PL) type II (Sigma, EC 3.1.1.3), suspended in Tris–HCl buffer (0.1 M Tris–HCl, pH 7.0 with 5mM CaCl_2_,), were preincubated at 37 °C for 15 min. Then, 20 μL p-NPB substrate (10 mM in DMSO) were added and incubated at 37 °C for 15 min. The absorbance was measured at 405 nm. Orlistat, a known PL inhibitor, was used as a positive control. Lipase inhibition was determined according to the Equation (1).

### 3.5. Antibacterial Activity

#### 3.5.1. Microorganisms and Culture Conditions

The bacterial strains tested with the WSE were *Staphylococcus aureus* (ATCC 49444) (Gram-positive) and *Escherichia coli* (ATCC 25922), *Pseudomonas aeruginosa* (ATCC 27853), and *Salmonella enteritidis* (ATCC 13076) (Gram-negative bacteria). All the microorganisms were obtained from the University of Agricultural Sciences and Veterinary Medicine (Food Biotechnology Laboratory) Cluj-Napoca, Romania. The strains were cultured on Muller-Hinton (MH) Agar and cultures were stored at 4 °C.

#### 3.5.2. Microdilution Method

A modified microdilution method was used to evaluate antimicrobial activity. Bacterial species were cultured overnight at 30–37 °C in Tryptic Soy Broth (TSB) and Muller-Hinton Broth (MHB). Suspensions of the microorganisms were adjusted with sterile saline in order to contain approximately 3 × 10^5^ colony forming units (CFU)/mL in a final volume of 100 µl per well. For further use, the inoculum was stored at 4 °C. Dilutions of the inoculum were cultured on MH to verify the absence of contamination and to check the validity of the inoculum. The minimum inhibitory concentrations (MICs) were assayed using 96-well microtitre plates. Different extract dilutions were mixed with 100 μL of TSB/MHB and 10 μL of inoculum, followed by incubation at 37 °C for 18 h. The MICs of the samples were detected following the addition of 20 μL (0.2 mg/mL) of resazurin solution to each well and incubation at 37 °C for 2 h. A change in color from blue to pink indicates resazurin reduction, hence bacterial growth. The lowest concentration that prevented this color change indicated the MIC. The antibiotic gentamycin (25 µL/well at a concentration of 4 µg/mL) was used as positive control and a solution of acetone in water (50%, *v/v*) was used as negative control. All determinations were performed in triplicate, and values are the averages of three replicates.

### 3.6. Antifungal Activity

The fungi used for this bioassay, *Candida albicans* (ATCC 10231) and *Candida parapsilosis* (ATCC 22019), were obtained from the same source as mentioned above. A microdilution method [34] was employed to assay the antifungal activity. Cultures were maintained on malt agar at 4 °C and sub-cultured every month. Suspensions of spores (1.0 × 10^5^ CFU/mL) was obtained by washing agar plates with sterile solution (0.85% saline and 0.10% Tween 80 (*v/v*)), in a final well volume of 100 µL. The inoculum was cultured on a solid Malt Broth (MB) and screened for contamination. The MICs were determined using a serial of dilutions in 96-well plates. Different dilutions in 0.85% saline (10 mg/mL) were mixed with MB and inoculum and incubated at 28 °C on a rotary shaker for 72 h. The lowest concentration without microscope visible growth was defined as MIC. The fungicide fluconazole was used as positive control (1–3500 µg/mL). All the experiments were performed in triplicate.

### 3.7. Antimutagenic Assay

Microbial mutagenicity was analyzed by the standard Ames test (standard plate incorporation assay) [65]. A preliminary toxic dose range trial was accomplished for the determination of an appropriate dose range for the test. This was done in order to avoid an inaccurate result caused by antimicrobial effect of tested samples toward the tester strains. Samples were prepared with 0.1 mL test strain cultured for 10 h (about 1 × 10^8^ cells/mL), 0.1 mL of extract (5 mg/plate), 0.1 mL of phosphate buffer (PB) (0.2 M, pH 7.4), and 0.5 mL of metabolic activation (S-9) mix or PB. The serial dilutions were immediately prepared with PB, and then 1 mL of the aliquot and 12 mL of nutrient agar were added. After incubation at 37 °C for 48 h, the colonies were counted. A toxicity effect was established if the standard plate count of the tested sample was lower than that of the control (with no compound added).

A mixture, containing 0.1 mL of extract (5 mg/plate) with 0.5 mL of S9 mix or PB, 0.2 mL of 0.5 mM histidine-biotin, and 0.1 mL test strain cultured for 10 h (approximately 10^8^ cells/mL), was added to a tube containing 2 mL of top agar. The tube was lightly vortexed and poured onto the medium agar (MA) plate. After incubating at 37 °C for 48 h, the number of revertant colonies was counted. The samples were assayed using triplicate plates. 4-Nitro-o-phenylenediamine (4-NPD) and sodium azide (NaN_3_) were used as positive control chemicals for *Salmonella typhimurium* strains TA 98 and TA 100, respectively. A compound was considered as a mutagen if there was a two-fold increase in the number of revertants, comparing with the number of spontaneous revertants (negative control) or a dose-related increase in the number of revertants for one or more strains. The mutagenicity of the diagnostic mutagens (positive control) in the absence of the extract was defined as 0% inhibition, and the antimutagenic effect was calculated according to the Equation (2):

(2) Inhibition (%) = (1 − T/M) × 100

where T is the number of revertants per plate in the presence of mutagen and the extract and M is the number of revertants per plate in the positive control. The tests were performed in duplicate with three sub-samples each, and the data was presented as mean ± standard deviation (SD).

### 3.8. Biological Activities on Cell Lines

#### 3.8.1. Cell Cultures

The human lung adenocarcinoma A549, the human breast cancer T47D-KBluc and MCF-7 cell lines were purchased from American Type Culture Collection (ATCC, Manassas, VA, USA). A549 and T47D-KBluc were maintained in Dulbecco’s Modified Eagle Medium (DMEM, Gibco, Paisley, UK) supplemented with 10% Fetal Bovine Serum (FBS, Sigma Aldrich, Steinheim, Germany), while the MCF-7 cell line was grown in phenol red-free DMEM/F12 medium supplemented with 1% L-glutamine, 1% sodium pyruvate, 1% MEM non-essential amino acids, 10 μg/mL insulin and 10% FBS. The normal human gingival fibroblasts (HGF) (CLS Cell Lines Service, Eppelheim, Germany) were maintained in DMEM supplemented with 10% FBS. All cells were routinely cultured in flasks at 37 °C in a humidified incubator with 5% CO_2_ supplementation, while the medium was changed every 2–3 days. The cells were used for the experiments or subcultured once they reached 70–80% confluence.

#### 3.8.2. Preparation of Extract Solutions

A 100 mg/mL stock solution of the septum extract was prepared in DMSO. The stock solution was diluted in DMSO to achieve working solutions of 0.25, 6.25, 12.5, 18.75, 25, 37.25, 50 and 75 mg/mL. These working solutions were then diluted in cell culture medium to obtain the concentrations selected for the in vitro experiments on cell lines. 

#### 3.8.3. Cytocompatibility

Cells were seeded in 96 well plates and left to attach for 24 h. Dead and unattached cells were removed by washing with PBS while the remaining viable cells were further exposed for 24 h/48 h to the WSE. Following the exposure, the medium was removed, the cells were washed with PBS and viability was measured by Alamar Blue (AB) assay. AB was used to measure the metabolic ability of exposed cells to convert resazurin, a non-fluorescent compound, to resorufin, a fluorescent product. The cells were exposed to a resazurin solution of 200 µM for 3–5 h and the fluorescence was measured at λ_excitation_ = 530/25; λ_emission_ = 590/35, using Synergy 2 Multi-Mode Microplate Reader (BioTek Instruments, Inc., Winooski, VT, USA). Three biological replicates, each one including six technical replicates were performed and included a negative control (cells exposed to culture medium containing 0.2% DMSO). The results were represented as relative values compared to the negative control (100%). To facilitate the interpretation of the results, when possible, IC_50_ values were calculated from the dose-effect curves obtained by fitting the experimental data with a 4-parameter logistic curve in SigmaPlot 11 software.

#### 3.8.4. Apoptosis Evaluation Using Flow Cytometry

Cells detached after trypsinization were collected, washed with phosphate buffer saline (PBS) which was previously cooled to 4 °C. The cell pellet was resuspended in binding buffer followed by the addition of Annexin-V FITC (Sigma, St. Louis, MO, USA) and incubated in the dark at 4 °C for 10 min. Propidium iodide (PI) was added to this mixture, resulting in a pale pink final coloration. Apoptosis assay was performed using a BD FACSCanto II flow cytometer (BD Biosciences, San Jose, CA, USA) and the data were analyzed with FACS Diva version 6.0 software.

#### 3.8.5. Antioxidant Potential

The ability of the WSE to mitigate the oxidative stress in A549, T47D-KBluc, MCF-7, and HGF-1 cells was evaluated using the reactive oxygen species (ROS) sensitive dye 2ʹ,7ʹ-Dichlorofluorescin Diacetate (DCFH-DA) as described previously [26]. All four cell types were treated for 24 h with three non-toxic Septum extract concentrations (25, 40, and 50 µg/mL). After the exposure, cells were washed gently with PBS and further incubated with 50 µM DCFH-DA in Hanks’ Balanced Salt Solution (HBSS) for 2 h. Subsequent to the loading, the excess of DCFH-DA was washed and the cells were exposed to 250 µM H_2_O_2_ or HBSS for 2 h in order to measure the quantity of ROS in stimulated and non-stimulated conditions. The conversion of DCFH-DA to the fluorescent compound dichlorofluorescein (DCF) was measured using Synergy 2 Multi-Mode Microplate Reader at λ_excitation_ = 485/20; λ_emission_ = 528/20. The potency of the Septum extract to mitigate the induction of oxidative stress after H_2_O_2_ treatment and in non-stimulated conditions was compared to N-Acetyl Cysteine (NAC) treatment (20 mM solution).

#### 3.8.6. Anti-Inflammatory Potential

The anti-inflammatory potential of the WSE was evaluated by measuring the levels of three pro-inflammatory cytokines, namely IL-6, IL-8, and IL-1β, in cell culture supernatant by ELISA assays. Normal gingival fibroblasts cells were concomitantly exposed to 100 ng/mL Lipopolysaccharides (LPS) from E. coli (Sigma Aldrich, Steinheim, Germany) and to three selected non-cytotoxic concentrations of the WSE for 24 h. Prior to the evaluation of the anti-inflammatory potential of the extract, the cellular viability was evaluated to exclude an additive cytotoxic effect of the extract in the presence of the LPS. Cell culture supernatants were harvested after a 24 h exposure and stored at −70°C until analysis. The concentrations of IL-8, IL-6, and IL-1β in cell culture supernatants were measured using commercially available ELISA Kits (EIAab Science Inc, China). The levels of sensitivity of the kits were 4.4 pg IL-8/mL, 1.6 pg IL-6/mL, and 0.15 pg IL-1β/mL. A cytokine standard curve was included in each experiment and cytokine levels were calculated from a four-parameter logistic curve according to the manufacturer’s instructions.

### 3.9. Statistical Analysis

All samples were analyzed in triplicate (*n* = 3), if not stated otherwise, and the outcomes were reported as the mean ± Standard Deviation (SD). The data were statistically analyzed by one-way ANOVA analysis of variance (with post-hoc Tukey Test for comparing multiple treatments) after normality and equal variance tests (Shapiro-Wilk) using SigmaPlot 11.0 computer software. The difference showing a p level of 0.05 or lower was considered statistically significant.

## 4. Conclusions

Food and agricultural industry by-products can represent valuable and inexpensive sources of bioactive compounds. Therefore, our study aimed to increase the knowledge regarding walnut septum, a by-product that currently has limited use.

The results of our work indicate that walnut septum can be a source of natural biologically active molecules. As far as we know, we determined for the first time the tocopherol content in this vegetable matrix. The tocopherols and the phenolics found in WSE present antioxidant and anti-inflammatory activities, or can prevent cholesterol and lipid peroxidation, therefore, significantly contribute to the valuable effects of walnut septum on health.

Further, our experiment investigated the in vitro effects of septum extract on some key enzymes involved in pathologies including neurodegenerative disorders, diabetes, and obesity. Walnut septum contains powerful α-glucosidase and lipase inhibitor phytochemicals that can obstruct dietary carbohydrate or lipid metabolism. Moreover, this is the first study to demonstrate that walnut septum extract presents antimicrobial and antimutagenic potential, as well as strong antioxidant and anti-inflammatory activities. Therefore, using septum as a source of phytochemicals can lead to the valorization of this by-product and may increase the value of walnut production.

## Figures and Tables

**Table 1:** Detection parameters for tocopherols determined by liquid chromatographic method coupled with mass spectrometry in tandem (LC-MS/MS).

Compound	R_T_ (min)	M	[M − H^+^]	*m/z* Ions
**δ** **-tocopherol**	3.3	402.6	401.6	401→386
**γ-tocopherol**	4.1	416.7	415.7	415→400
**α-tocopherol**	5.1	430.7	429.7	429→163

R_T_ – retention time.

**Table 2:** Tocopherol content (mg/100 g extract ± SD (standard deviation)) determined in walnut septum extract (WSE) and walnut kernel (with skin) extract (WE) by LC-MS/MS.

	α-Tocopherol	γ/β-Tocopherols	δ-Tocopherol	Total-Tocopherols
**WSE**	3.35 ± 0.04	1.73 ± 0.01	1.47 ± 0.02	6.55 ± 0.07
**WE**	0.78 ± 0.01	16.72 ± 0.63	2.06 ± 0.11	19.56 ± 0.74
**WSE/WE**	4.295	0.103	0.714	

WSE/WE – ratio of tocopherol content in WSE to that in WE.

**Table 3:** Phenolic compounds identified and quantified in the lyophilized WSE [4] (data not previously published).

Peak No.	Phenolic Compound	R_T_ (min)	*m/z*	Analysis (mg/100 g)
**1**	Gallic acid	1.5	169.1	7.96
**2**	Protocatechuic acid	2.8	153.1	0.99
**3**	Caftaric acid	3.5	311.2	˂ LOD
**4**	Gentisic acid	3.5	153.1	˂ LOQ
**5**	Caffeic acid	5.6	179.1	˂ LOD
**6**	Chlorogenic acid	5.6	353.3	˂ LOQ
**7**	Catechin	6.0	289.2	59.76
**8**	Vanillic acid	6.7	167.1	0.56
**9**	Syringic acid	8.4	197.2	0.52
**10**	Epicatechin	9.0	289.2	1.25
**11**	p-Coumaric acid	9.5	163.0	˂ LOQ
**12**	Ferulic acid	12.8	193.2	˂ LOQ
**13**	Sinapic acid	15.0	223.2	˂ LOD
**14**	Hyperoside	18.6	463.4	6.73
**15**	Isoquercitrin	19.6	463.1	10.36
**16**	Rutoside	20.2	609.5	˂ LOD
**17**	Myricetol	21.1	317.2	˂ LOD
**18**	Fisetin	22.9	285.2	˂ LOD
**19**	Quercitrin	23.6	447.4	107.3
**20**	Quercetin	26.8	301.2	˂ LOD
**21**	Patuletin	28.7	331.3	˂ LOD
**22**	Luteolin	29.1	285.2	˂ LOD
**23**	Kaempferol	32.5	285.2	˂ LOD
**24**	Apigenin	33.1	269.2	˂ LOD

LOD – limit of detection; LOQ – limit of quantification; R_T_ – retention time.

**Table 4:** Minimum Inhibitory Concentration (MIC) for the walnut septum extract.

Samples	*Staphylococcus aureus*	*Escherichia coli*	*Pseudomonas aeruginosa*	*Salmonella enteritidis*	*Candida albicans*	*Candida parapsilosis*
WSE (mg/mL)	0.098	1.56	0.012	0.098	3.12	3.12
Gentamycin/ Fluconazole (μg/mL)	0.038	1.2	1.2	2.4	0.1	0.1

WSE – walnut septum extract; each value is the mean of three independent measurements.

**Table 5:** Antimutagenity assay for *Salmonella typhimurium* TA 98 and TA 100 strains.

Test Item	Number of Revertants			
	TA 98		TA 100	
	Mean (±SD)	Inhibition (%)	Mean (±SD)	Inhibition (%)
**Negative Control**	9.25 ± 3.6	-	9.25 ± 2.4	-
**WSE**	124 ± 4.4	36.08	198 ± 6.3	43.27
**4-NPD/NaN_3_**	194 ± 3.3	0	349 ± 15.22	0

WSE – walnut septum extract. Positive controls: 4-NPD (4-Nitro-o-phenylenediamine) and NaN_3_ (sodium azide) for TA 98 and TA 100, respectively. SD—standard deviation.

**Table 6:** IC_50_ values (µg extract/mL) ± Standard Error obtained by Alamar Blue assay for A549, T47D-KBluc, MCF-7, and HGF at 24 h and 48 h post-exposure.

Cell Lines	Exposure Duration (h)	
	24 h	48 h
A549	80.02 ± 4.33	70.79 ± 1.93
T47D-KBluc	265.60 ± 53.79	112.75 ± 6.38
MCF-7	>400	>400
HGF	>400	254.25 ± 3.56

**Table 7:** The percentage of viable, necrotic, early apoptotic and late apoptotic cells (A549, T47D-KBluc) after a 24 h exposure to WSE, measured using flow cytometry.

Cell Type	Dose (µg/mL)	Viable Cells (%)	Necrotic Cells (%)	Early Apoptotic (%)	Late Apoptotic (%)
**A549**	NC	98.05 ± 0.95	0.43 ± 0.15	0.93 ± 0.57	0.53 ± 0.41
	100	58.23 ± 2.51 *	30.13 ± 5.10 *	6.1 ± 1.15 *	5.66 ± 2.53 *
	200	49.65 ± 3.63 *	41.66 ± 7.80 *	3.36 ± 0.55 *	5.8 ± 4.07 *
	400	28.96 ± 2.75 *	69.83 ± 2.65 *	0.03 ± 0.05	0.61 ± 0.26
**T47D-KBluc**	NC	98.30 ± 0.43	0.50 ± 0.17	0.63 ± 0.15	0.53 ± 0.40
	100	80.03 ± 5.12 *	12.13 ± 8.35 *	1.73 ± 1.79	6.03 ± 5.4
	200	42.73 ± 4.05 *	56.53 ± 4.18 *	0	0.40 ± 0.26
	400	12.45 ± 3.25 *	84.85 ± 3.75 *	0.1 ± 0.1	2.65 ± 1.45

The results are expressed as relative means ± standard deviations (three biological replicates). Asterisks (*) indicate significant differences (*p* < 0.05) compared to the negative control. NC—negative control.
